# Visual Acuity Outcomes in Diseases Associated with Reduced Visual Acuity: An Analysis of the National Health Insurance Service Database in Korea

**DOI:** 10.3390/ijerph19148689

**Published:** 2022-07-17

**Authors:** Sang-Yeob Kim, Byeong-Yeon Moon, Hyun-Gug Cho, Dong-Sik Yu

**Affiliations:** Department of Optometry, Kangwon National University, Samcheok 25949, Korea; syk@kangwon.ac.kr (S.-Y.K.); bymoon@kangwon.ac.kr (B.-Y.M.); hyung@kangwon.ac.kr (H.-G.C.)

**Keywords:** visual acuity, hypertension, diabetes mellitus, glaucoma, diabetic retinopathy

## Abstract

Visual acuity declines with age, and disease-related visual acuity changes vary. We evaluated factors affecting visual acuity and age-related visual acuity in diseases associated with reduced visual acuity such as hypertension, diabetes mellitus (DM), glaucoma, and diabetic retinopathy (DR). The Korean National Health Insurance Service 2015–2016 data were analyzed for age-related visual acuity changes and prevalence of diseases associated with reduced visual acuity. Among 993,062 participants, the prevalence rates of hypertension, DM, glaucoma, and DR were 27.0%, 15.1%, 13.8%, and 2.7%, respectively. Despite having the lowest prevalence, DR alone or DR with hypertension and glaucoma resulted in low visual acuity. Correlation analysis between disease frequency and mean age-related visual acuity revealed higher positive correlations in DR and hypertension than in DM and glaucoma, indicating lower visual acuity. Odds ratios for low visual acuity in cases including one disease such as hypertension, DM, glaucoma, and DR were 1.73, 1.23, 1.04, and 1.52, respectively. The prevalence and number of diseases associated with reduced visual acuity increased with age, and visual acuity decreased. The leading causes of vision loss were DR as a single disease and hypertension as a concomitant disease. Therefore, age-related vision management, through periodic eye examination and correction with age, should be performed along with management of diabetes and hypertension.

## 1. Introduction

Visual acuity evaluation is important for detecting changes in vision that are related to eye health and quality of life [1,2]. To maintain healthy vision for a lifetime, individuals should undergo periodic eye examinations. In South Korea, almost everyone undergoes various eye examinations for preschool or school vision screening [3,4], employment [5], military service [6], and health care [7]. Moreover, all people residing in Korea are obliged to enroll in the National Health Insurance Service (NHIS), a social and health security system. All people enrolled in the NHIS are required to undergo a health checkup, including a visual acuity test, biannually (annually for non-office workers). In addition, the Korea National Health and Nutrition Examination Survey (KNHANES) has been investigating the health and nutritional status of Koreans since 1998 [8]. The KNHANES conducts periodic surveys of 10,000 individuals aged ≥ 1 year, and currently, KNHANES VIII (2019–2021) is in progress [9].

For the evaluation of eye health, the NHIS program focuses on visual acuity assessments at hospitals, as part of primary eye care for all citizens, while the KNHANES program focuses on the detection of eye-related diseases through ophthalmic interviews and examinations conducted in households. Both the NHIS and KNHANES provide data for the development and evaluation of health policies in Korea. These data have enabled population-based studies on cataract, glaucoma, age-related macular degeneration (AMD), and diabetic retinopathy (DR) in Korea and many other countries [7,10,11,12,13,14]. Although the NHIS provides extensive medical services compared to similar systems in other countries, only a few NHIS data-based studies on vision have been conducted [15,16].

According to the World Health Organization [17], the leading causes of vision impairment globally are uncorrected refractive errors, cataract, AMD, glaucoma, and DR. However, the main causes vary across countries and depend on the availability of health and eye care services [13]. In Korea, the prevalence of cataracts is increasing; however, they rarely cause blindness owing to the development and utilization of appropriate medical treatment services [13,18]. The three major causes of blindness and vision impairment are DR, AMD, and glaucoma [19]. Two diseases related to visual acuity, other than AMD, that are regarded as high-risk factors are hypertension and diabetes mellitus (DM) [14,20]. AMD is considered as the primary cause of blindness in the elderly; however, it is also influenced by genetic factors [21]. Therefore, the NHIS in Korea emphasizes the importance of managing diseases associated with reduced visual acuity to prevent and control blindness and vision impairment and provides data on visual acuity and diseases associated with reduced visual acuity such as hypertension, DM, glaucoma, and DR [22]. In Korea, the prevalence rates of these diseases were 38.5% for hypertension among adults ≥ 30 years in 2015 [10], 14.4% for DM among adults ≥ 30 years in 2016 [11], 15.9% for DR in patients with DM ≥ 30 years in 2015 [12], and 1.05% for glaucoma among adults ≥ 40 years based on the NHIS in 2013 [23]. The prevalence rates of diseases associated with reduced visual acuity are increasing annually; aging also leads to increase in prevalence of these diseases.

This study was designed with a focus on systemic diseases such as hypertension and DM that cause glaucoma and DR. The purpose of this study was to investigate age-related visual acuity changes, differences in visual acuity of people affected with diseases and the non-affected people, comparison of visual acuity among diseases, and risks of visual acuity based on the NHIS data.

## 2. Materials and Methods

### 2.1. Data Source and Study Population

**Figure 1:**
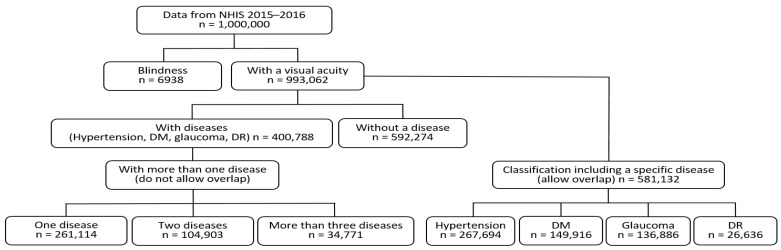
Flow chart of the study population. NHIS, National Health Insurance Service; DM, diabetes mellitus; DR, diabetic retinopathy.

The NHIS in Korea operates the National Health Insurance Sharing Service (nhiss.nhis.or.kr) to provide medical services and develop related policies by reporting various data sources such as sample research, customized, and health disease index databases. The data (released for the first time on 24 November 2021) used in the present study were obtained from the National Health Insurance Sharing Service for one million adults aged ≥ 20 years during 2015–2016 [22]. The data mainly included visual acuity and a history of diagnostic tests for hypertension, DM, glaucoma, and DR. The flow chart of the study population according to diseases is shown in Figure 1. This study was exempted from full review by the Institutional Review Board of Kangwon National University (KWNUIRB-2021-07-010) because the data source comprised de-identified secondary data released to the public for research purposes.

### 2.2. Data Variables

The age of the participants was stratified into 27 groups at 2-year intervals, except for the first group that included participants aged between 20–24 years and the last group that comprised those aged ≥ 75 years. The median value of each interval was used to evaluate the effect of age. We evaluated visual acuity of both eyes because significant differences might exist in older populations with diseases associated with reduced visual acuity. Visual acuity variables for the right and left eyes were expressed as decimal visual acuity ranging from 0.1 to 2.0; values of 9.9 (as a nominal value) for blindness and 0.1 for values less than 0.1 were excluded from the evaluation of visual acuity. To investigate the effect of visual acuity, decimal visual acuity was converted to logarithm of the minimum angle of resolution (logMAR) visual acuity using -log (decimal acuity), and lower logMAR values corresponded to better visual acuity. We also classified the presence or absence of disease as a nominal variable, i.e., the history of diagnostic tests for hypertension, DM, glaucoma, and DR, based on whether a disease diagnosis test was performed between 2012 and 2016. Hypertension included essential (primary) hypertension, hypertensive heart disease, hypertensive renal disease, hypertensive heart and renal disease, and secondary hypertension. DM included type 1 DM, type 2 DM, malnutrition-related DM, other specified DM, and unspecified DM. Glaucoma included glaucoma suspect (ocular hypertension), primary open-angle glaucoma, primary angle-closure glaucoma, glaucoma secondary to eye diseases, and glaucoma in diseases classified elsewhere. DR included type 1 and 2 DM, malnutrition-related DM, other specified DM, and unspecified DM, with diabetic non-proliferative or proliferative retinopathy or with other and unspecified retinopathies [22]. Although AMD is a disease that affects visual acuity, it was excluded from this study because it was not provided in the database.

### 2.3. Statistical Analysis

All data were statistically analyzed using IBM SPSS Statistics for Windows, Version 26.0 (IBM Corp., Armonk, NY, USA). The Kolmogorov–Smirnov test was used to assess for normality. The independent- samples *t*-test was used to compare the mean visual acuity between participants with and without diseases. The paired *t*-test was performed to evaluate the mean visual acuity between the right and left eyes. One-way analysis of variance (ANOVA) followed by Bonferroni’s post hoc test was performed to compare visual acuity according to diseases associated with reduced visual acuity. Changes in age-related visual acuity and diseases were evaluated using linear regression analysis. Diseases associated with a low visual acuity (less than 0.3 decimal acuity as low vision) were represented using the crude odds ratio (OR) in the unadjusted multivariate binary logistic regression analysis. The ORs and 95% confidence intervals (CIs) are also presented. A *p*-value less than 0.05 in all analyses was considered statistically significant.

## 3. Results

### 3.1. Diseases Associated with Reduced Visual Acuity and Visual Acuity

This study included 993,062 (99.3%) participants with visual acuity data (mean age ± standard deviation, 48.6 ± 14.1 years; males, 50.9%), excluding 6938 (0.7%) participants with blindness or zero decimal visual acuity.

Table 1 shows the characteristics of the study participants and the distribution (or frequency as a percentage) of diseases associated with reduced visual acuity. The proportions of participants with and without disease were 59.6% and 40.4%, respectively. Among the 993,062 participants, 27.0% had hypertension, 15.1% had DM, 13.8% had glaucoma, and 2.7% had DR. Among 400,788 participants with ≥one disease, 65.2%, 26.2%, and 8.7% had 1, 2, and ≥3 diseases, respectively. Among the single diseases, hypertension, glaucoma, DM, and DR showed the highest distribution in the given order. However, in cases of a specific disease, among the 400,788 participants, 66.8% had hypertension, 37.4% had DM, 34.3% had glaucoma, and 6.6% had DR. This result showed a higher distribution of DM than that of glaucoma, unlike in cases of single diseases.

Table 2 shows comparisons of visual acuity in different classifications of diseases associated with reduced visual acuity. Participants with ≥one disease had lower visual acuity than those without a disease (independent-samples *t*-test, *p* < 0.001). In participants with one disease, logMAR visual acuity for participants with hypertension (0.12 ± 0.22 for both eyes) or DR (0.12 ± 0.26 and 0.12 ± 0.25 for the right and left eyes, respectively) was lower than that for participants with DM (0.10 ± 0.21 for both eyes) or glaucoma (0.06 ± 0.20 for both eyes) (one-way ANOVA, *p* < 0.001). In participants with two diseases, hypertension (0.18 ± 0.25 and 0.19 ± 0.26 for the right and left eyes, respectively) and glaucoma, including DR (0.17 ± 0.28 and 0.15 ± 0.26 for the right and left eyes, respectively) resulted in low visual acuity (one-way ANOVA, *p* < 0.001). In participants with more than three diseases, those with hypertension, glaucoma, and DR (0.23 ± 0.28 and 0.22 ± 0.27 for the right and left eyes, respectively) and those with all the four diseases (0.21 ± 0.26 and 0.21 ± 0.26 for the right and left eyes, respectively) had low visual acuity. When visual acuity, according to the number of diseases, was compared in all cases, visual acuity decreased as the number of diseases increased. In case of a specific disease, visual acuity was lower for DR, followed by DM, hypertension, and glaucoma (one-way ANOVA, *p* < 0.001). Cases of a specific disease had lower visual acuity than cases of a single disease. Considering all the above cases, visual acuity related to DR was lower than that related to other diseases. However, the differences in visual acuity between the right and left eyes according to diseases associated with reduced visual acuity were not significant (paired *t*-test, *p* > 0.05).

### 3.2. Linear Regression Analysis

**Figure 2:**
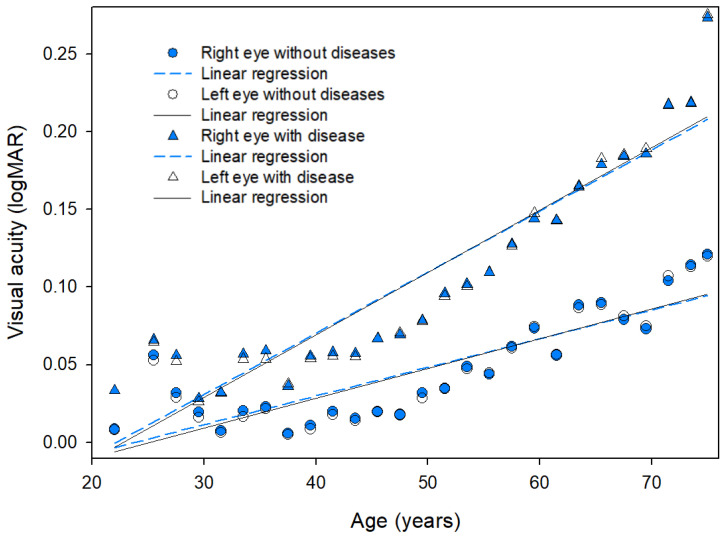
Linear regression for age and mean visual acuity.

Linear regression analysis to estimate changes in visual acuity and diseases with age was performed on data from 400,788 participants with ≥one disease. The equations used for the regression analysis for age (*x*-axis) and mean visual acuity (*y*-axis) are shown in Figure 2. The association between the two variables showed a high linear correlation, y = 0.002x − 0.041 (R^2^ = 0.699, *p* < 0.001) for the right eye and y = 0.002x − 0.046 (R^2^ = 0.733, *p* < 0.001) for the left eye in patients without diseases; in those with diseases, the correction was as follows: y = 0.004x − 0.084 (R^2^ = 0.862, *p* < 0.001) for the right eye and y = 0.004x − 0.096 (R^2^ = 0.875, *p* < 0.001) for the left eye. Linear regression equations for bilinear models with a breakpoint based on 30, 36, 40, 46, and 50 years between age and visual acuity were not obtained.

The results of the linear regression analysis between age (*x*-axis) and disease (*y*-axis) are shown in Table 3. Although all the coefficients of determination were significant, age was more significant with the number of diseases (R^2^ = 0.235, *p* < 0.001) than with each disease (R^2^ = 0.003 − 0.036, *p* < 0.001).

### 3.3. Correlation Analysis between the Frequency of Diseases and Age-Related Visual Acuity

**Figure 3:**
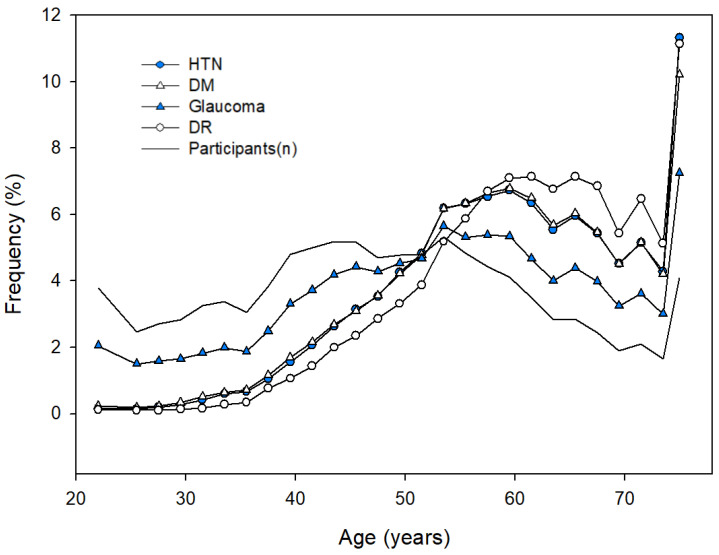
Frequency of diseases with age. DM, diabetes mellitus; DR, diabetic retinopathy; HTN, hypertension.

The frequency (%) of disease with age based on equal weights in 400,788 participants with ≥one disease is shown in Figure 3. The Pearson correlation coefficient between each disease and the number of participants with age was not significant, except in glaucoma (r = 0.513, *p* = 0.006). However, the Pearson correlation coefficient among each disease was significant (from r = 0.918 for DM vs. glaucoma to r = 0.997 for hypertension vs. DM, *p* < 0.001).

The Pearson correlation coefficient between each disease and mean visual acuity was significant, as shown in Table 4. The frequency of diseases and age-related visual acuity showed a high positive correlation in the following order: DR (r = 0.907 and 0.891 for the right and left eyes, respectively), hypertension (r = 0.826 and 0.810 for the right and left eyes, respectively), DM (r = 0.818 and 0.802 for the right and left eyes, respectively), and glaucoma (r = 0.577 and 0.556 for the right and left eyes, respectively). In the linear regression equation between age and frequency of diseases, as shown in Table 5, the coefficient of determination was significant, with R^2^ = 0.823 for DR, 0.721 for DM, 0.701 for hypertension, and 0.393 for DR (*p* < 0.001).

### 3.4. Diseases Associated with Low Visual Acuity

In the unadjusted multivariate binary logistic regression analysis, the ORs of low visual acuity of less than 0.3 decimal acuity (0.52 logMAR) were 0.98 for DR and lower for other diseases in cases of a single disease, and 1.73 for hypertension, 1.52 for DR, and higher than 1.00 for other diseases in cases including one disease, as shown in Table 6.

## 4. Discussion

In this study, we evaluated disease-related visual acuity in a large population of participants aged ≥ 20 years using data on visual acuity and characteristics of four diseases, including hypertension, DM, glaucoma, and DR, obtained from the NHIS in Korea. The main findings of our study are as follows: a high prevalence of hypertension resulted in low visual acuity, and hypertension and glaucoma with DR resulted in low visual acuity; the higher the number of diseases, the lower the visual acuity; DR was a primary factor contributing to poor visual acuity; each disease and the number of diseases increased slightly with age; visual acuity decreased with age in DM, hypertension, and especially DR rather than in glaucoma; and age-related low visual acuity was significantly affected by hypertension and DR.

In the present study based on NHIS data, the prevalence of hypertension was 27.0%, which was similar to the prevalence of 28.1% reported for adults aged ≥ 19 years in 2019 by the Korea Statistical Information Service [24] and somewhat higher than the 21% reported for the whole Korean population [25]. Moreover, the prevalence of DM was 15.1%, which was similar to the prevalence of 14.5% reported for adults aged ≥ 30 years and 12.2% reported for those aged ≥ 19 years in 2019 by the Korea Statistical Information Service [24]. The prevalence of DM was close to double of 8.6% reported for the Korean population aged ≥ 20 years in the 2021 International Diabetes Federation Diabetes Atlas [26]. Further, the prevalence of glaucoma was 13.8%, which was higher than the 1.05–4.5% reported in Korea [27,28,29] and the 3.53% reported by another study [30]. The high prevalence reported in our study may be related to the fact that the number of patients with glaucoma increased on average by 9% annually between 2009 and 2019 [23,28]. However, the prevalence of DR was 2.7%, which was within the range of 2–4.5% reported by a systematic review [31]. Therefore, the prevalence of a single disease was more frequent than multiple diseases. In addition, DM was less frequent than glaucoma as a single disease, but more frequent in the case of multiple diseases. A high prevalence of DR (16%) in patients with DM has been reported previously [32]. Therefore, our findings confirmed the coexistence of DM with other diseases, such as hypertension [33].

Several studies have reported associations between vision loss and diseases such as hypertension, DM, glaucoma, and DR [33,34,35,36]. Our evaluation of visual acuity related to diseases confirmed that these diseases were vision-related factors, and the more the number of diseases, the lower the visual acuity. Despite having the lowest frequency, DR resulted in low visual acuity both as a single disease and when concomitant with multiple diseases such as hypertension and glaucoma [34]. Among the diseases associated with reduced visual acuity analyzed in this study, DR was the leading cause of poor visual acuity and vision loss in adults globally [37,38].

Early studies on age-related changes in decimal visual acuity [39,40] showed that older adults without a specific visual pathology exhibited an age-related decrease in visual acuity, similar to individuals with documented visual pathologies. However, the above studies had limitations as they used decimal visual acuity instead of logMAR visual acuity, included populations of only tens or hundreds of individuals with glaucoma and cataract or age-related maculopathy, and did not clearly reveal the difference between visual acuity with and without diseases. Unlike these studies, ours was based on a large population, utilized logMAR visual acuity, and analyzed four diseases. Moreover, our findings based on linear regression analyses revealed that the visual acuity outcomes according to age tended to be worse with diseases than without diseases in terms of the coefficient of correlation. Other studies performing regression analysis of ocular refraction or age-related visual acuity [41,42] showed that visual acuity of healthy eyes and ocular refractive errors related to visual acuity had a point of inflection around the age of 30 years. However, bilinear models with a breakpoint based on 30–50 years of age and visual acuity were not included in our study. Although our findings revealed no trend in bilinear models until the age of 50 years, the above analyses revealed little reduction in visual acuity before 50 years of age.

In this study, the prevalence of hypertension, DM, glaucoma, and DR increased slightly with age, and the number of diseases increased moderately. Various trends in the age-related prevalence of these diseases have been reported in the literature [43,44,45]. However, in our analysis of the frequency of diseases according to age, the frequency of each disease was not significantly correlated with the number of participants, except in the case of glaucoma. However, a very high positive correlation of 0.918 or more was observed between each disease. These results suggest that the frequency of a disease correlates more with the frequency of other diseases than with the percentage of participants. The association between diseases has been reported in several previous studies, including the association of hypertension with DR [14], presence and risk factors for glaucoma in patients with DM [33], the prevalence of DR in DM [27], and an association between DM and glaucoma [36].

We attempted to estimate whether the number of diseases increases with age through linear regression analysis. Although age had a very low explanatory power for the increasing number of diseases, there was a weak relationship. In the correlation analysis between the frequency of diseases and mean age-related visual acuity, a higher positive correlation between DR and hypertension than between DM and glaucoma indicated lower visual acuity. In addition, the frequency of diseases with age was higher in DR, DM, and hypertension than in glaucoma. The frequency of diseases increased with increasing age, and visual acuity decreased with increasing age in DM, hypertension, and especially DR than in glaucoma. Until recently, no studies have compared visual acuity among DR, hypertension, DM, and glaucoma. Our study revealed that visual acuity was lower in DR, followed by hypertension, DM, and glaucoma. The lower visual acuity in DR than in other diseases may be attributed to the fact that it is a disease affecting the retina, which is directly related to visual acuity [46]. In contrast, visual acuity was relatively higher in glaucoma than in other diseases, possibly because glaucoma affects the visual field rather than visual acuity; contrast sensitivity was reported to be more relevant than visual acuity in patients with early changes in glaucoma [47].

The ORs for low visual acuity according to diseases associated with reduced visual acuity showed a higher risk in individuals with two diseases than in those with a single disease, and age-related low visual acuity was largely influenced by hypertension and DR. The high influence of hypertension and DR may be related to their interactions, as shown in a recent study in which both poorly controlled and untreated hypertension were significantly associated with DR showing ORs of 1.97 and 2.01, respectively [14].

The strengths of this study are as follows: this study, for the first time, included and analyzed a large group of data collected from 97% of the population enrolled in the Korean NHIS, and focused on diseases associated with reduced visual acuity in individuals with a wide age range of ≥20 years. Nevertheless, there are limitations to the raw data that might affect the visual acuity outcomes for diseases associated with reduced visual acuity. First, the scale intervals of visual acuity [23] were different because of data measurement based on decimal visual acuity. Second, visual acuity lower than 0.1 was unknown because the lowest visual acuity was 0.1. Third, the severity of diseases was unknown. In the presence of multiple diseases such as hypertension and diabetes, an ophthalmological assessment of the causes of reduced visual acuity could not obtained in detail. Lastly, the study was limited to evaluating only visual acuity, which is only one among many visual functions used for evaluating vision. To overcome these limitations, we used logMAR visual acuity instead of decimal visual acuity, and visual acuity less than 0.1 was not considered. Nonetheless, our results are likely to be more meaningful because these limitations tend to result in underestimation rather than overestimation.

## 5. Conclusions

This study involves visual acuity data related to hypertension and diabetes mellitus of a very large group of subjects based on the NHIS in Korea. Visual acuity decreased with age, and the prevalence of diseases such as hypertension, DM, glaucoma, and DR increased with age. Participants with history of a disease diagnosis had lower visual acuity. Visual acuity was also lower in those with a history of multiple disease diagnosis than in those with a single disease diagnosis. Moreover, visual acuity was lower in DR as a single disease and in hypertension as one of the multiple diseases. Therefore, we suggest that age-related vision management, such as periodic eye examination and correction with age, should be more closely related to diabetes and hypertension management than glaucoma and DR management.

## Figures and Tables

**Table 1:** Participant characteristics and distribution of diseases associated with reduced visual acuity.

Participant Characteristics	*n*	%
** All participants **	1,000,000	
With visual acuity	993,062	99.3
Blindness	6938	0.7
** Participants with visual acuity **	993,062	
Without a disease	592,274	59.6
With ≥one disease	400,788	40.4
** Participants with ≥one disease **	400,788	
Hypertension	144,687	36.1
DM ^1^	41,527	10.4
Glaucoma	74,246	18.5
DR ^2^	654	0.2
Hypertension + DM	60,184	15.0
Hypertension + glaucoma	29,798	7.4
Hypertension + DR	617	0.2
DM + glaucoma	8642	2.2
DM + DR	5230	1.3
Glaucoma + DR	432	0.1
Hypertension + DM + glaucoma	15,068	3.8
Hypertension + DM + DR	11,003	2.7
Hypertension + glaucoma + DR	438	0.1
DM + glaucoma + DR	2363	0.6
Hypertension + DM + glaucoma + DR	5899	1.5
** Participants including vs. excluding a disease **	993,062	
Included vs. excluded hypertension	267,694 vs. 725,368	27.0 vs. 73.0
Included vs. excluded DM	149,916 vs. 843,146	15.1 vs. 84.9
Included vs. excluded glaucoma	136,886 vs. 856,176	13.8 vs. 86.2
Included vs. excluded DR	26,636 vs. 966,426	2.7 vs. 97.3

^1^ diabetes mellitus; ^2^ diabetic retinopathy.

**Table 2:** Mean and standard deviation visual acuity according to diseases associated with reduced visual acuity.

Classification	Right Eye	Left Eye	*p*-Value (Post-Hoc)
Without a disease	0.04 ± 0.19	0.04 ± 0.19	<0.001 for the independent-samples *t*-test
With ≥one disease	0.12 ± 0.22	0.12 ± 0.22	
** A. Including only one disease **	0.10 ± 0.21	0.10 ± 0.21	0.374 for the paired *t*-test
Hypertension	0.12 ± 0.22	0.12 ± 0.22	<0.001 for one-way ANOVA ^3^ (Hypertension, DR > DM > glaucoma)
DM ^1^	0.10 ± 0.21	0.10 ± 0.21	
Glaucoma	0.06 ± 0.20	0.06 ± 0.20	
DR ^2^	0.12 ± 0.26	0.12 ± 0.25	
** B. Including two diseases **	0.14 ± 0.22	0.14 ± 0.22	0.074 for the paired *t*-test
(a) Hypertension + DM	0.15 ± 0.22	0.15 ± 0.22	<0.001 for one-way ANOVA (a, b, c, e, f > d)
(b) Hypertension + glaucoma	0.14 ± 0.23	0.14 ± 0.23	
(c) Hypertension + DR	0.18 ± 0.25	0.19 ± 0.26	
(d) DM + glaucoma	0.12 ± 0.21	0.12 ± 0.21	
(e) DM + DR	0.14 ± 0.22	0.13 ± 0.22	
(f) Glaucoma + DR	0.17 ± 0.28	0.15 ± 0.26	
** C. Including three or more diseases **	0.19 ± 0.24	0.18 ± 0.24	0.123 for the paired *t*-test
(a) Hypertension + DM + glaucoma	0.17 ± 0.23	0.17 ± 0.23	<0.001 for one-way ANOVA (b, c, e > a, d)
(b) Hypertension + DM + DR	0.19 ± 0.24	0.19 ± 0.24	
(c) Hypertension + glaucoma + DR	0.23 ± 0.28	0.22 ± 0.27	
(d) DM + glaucoma + DR	0.16 ± 0.23	0.16 ± 0.23	
(e) Hypertension + DM + glaucoma + DR	0.21 ± 0.26	0.21 ± 0.26	
** D. Including the following diseases **	0.13 ± 0.23	0.13 ± 0.22	0.675 for paired *t*-test
Hypertension	0.14 ± 0.22	0.14 ± 0.22	<0.001 for one-way ANOVA (DR > DM > hypertension > glaucoma, DM − hypertension = 0.004)
DM	0.14 ± 0.22	0.14 ± 0.22	
Glaucoma	0.10 ± 0.22	0.10 ± 0.22	
DR	0.18 ± 0.24	0.18 ± 0.24	
** Comparison of visual acuity among A, B, C, and D **	<0.001 for one-way ANOVA (C > B > D > A)		

Visual acuity is the logarithm of the minimum angle of resolution (logMAR) visual acuity. ^1^ diabetes mellitus; ^2^ diabetic retinopathy; ^3^ analysis of variance.

**Table 3:** Linear regression equation between age and number of diseases.

	Linear Regression Equation	R^2^	*p*-Value
Age vs. number of diseases	y = 0.032x − 0.962	0.235	<0.001
Age vs. hypertension	y = 0.006x − 0.324	0.036	<0.001
Age vs. DM ^1^	y = 0.003x − 0.213	0.007	<0.001
Age vs. glaucoma	y = 0.002x − 0.470	0.005	<0.001
Age vs. DR ^2^	y = 0.001x − 0.010	0.003	<0.001

^1^ diabetes mellitus; ^2^ diabetic retinopathy.

**Table 4:** Correlation between the frequency of diseases and mean visual acuity.

Visual Acuity	Hypertension	DM ^1^	Glaucoma	DR ^2^
Right eye	0.826 (*p* < 0.001)	0.818 (*p* < 0.001)	0.577 (*p* < 0.001)	0.907 (*p* < 0.001)
Left eye	0.810 (*p* < 0.001)	0.802 (*p* < 0.001)	0.556 (*p* < 0.001)	0.891 (*p* < 0.001)

Visual acuity is measured using the logarithm of the minimum angle of resolution (logMAR). ^1^ diabetes mellitus; ^2^ diabetic retinopathy.

**Table 5:** Linear regression equation between age and the frequency of diseases.

	Linear Regression Equation	R^2^	*p*-Value
Age vs. hypertension	y = 0.147x − 3.636	0.701	<0.001
Age vs. DM ^1^	y = 0.140x − 3.318	0.721	<0.001
Age vs. glaucoma	y = 0.056x + 0.827	0.393	<0.001
Age vs. DR ^2^	y = 0.177x − 5.067	0.823	<0.001

^1^ diabetes mellitus; ^2^ diabetic retinopathy.

**Table 6:** Odds ratios for low visual acuity according to diseases associated with reduced visual acuity.

	*n*	Crude Odds Ratio	95% CI ^1^	*p*-Value
Including only one disease	261,114			
Hypertension	144,687	0.83	0.79–0.86	<0.001
DM ^2^	41,527	0.61	0.57–0.65	<0.001
Glaucoma	74,246	0.47	0.44–0.49	<0.001
DR ^3^	654	0.98	0.66–1.45	0.918
Including the following diseases	581,132			
Hypertension	267,694	1.73	1.65–1.80	<0.001
DM	149,916	1.23	1.18–1.28	<0.001
Glaucoma	136,886	1.04	1.00–1.08	0.076
DR	26,636	1.52	1.43–1.61	<0.001

^1^ Confidence interval; ^2^ diabetes mellitus; ^3^ diabetic retinopathy.

## Data Availability

The data presented in this study are available at the National Health Insurance Service (https://nhiss.nhis.or.kr/bd/ab/bdabf010cv.do) (accessed on 1 July 2021).
